# Motivational salience and genetic variability of dopamine D2 receptor expression interact in the modulation of interference processing

**DOI:** 10.3389/fnhum.2013.00250

**Published:** 2013-06-05

**Authors:** Anni Richter, Sylvia Richter, Adriana Barman, Joram Soch, Marieke Klein, Anne Assmann, Catherine Libeau, Gusalija Behnisch, Torsten Wüstenberg, Constanze I. Seidenbecher, Björn H. Schott

**Affiliations:** ^1^Department of Behavioral Neurology and Department of Neurochemistry and Molecular Biology, Leibniz Institute for NeurobiologyMagdeburg, Germany; ^2^Department of Clinical Psychology, University of SalzburgSalzburg, Austria; ^3^Department of Psychiatry, Otto von Guericke University of MagdeburgMagdeburg, Germany; ^4^Department of Cognitive Neuroscience, Donders Institute for Brain, Cognition and Behaviour, Radboud University Nijmegen Medical CenterNijmegen, Netherlands; ^5^Department of Genetics, Radboud University Nijmegen Medical CentreNijmegen, Netherlands; ^6^Department of Neurology, Otto von Guericke University of MagdeburgMagdeburg, Germany; ^7^Department of Psychiatry, Campus Mitte, Charité University HospitalBerlin, Germany; ^8^Center for Behavioral and Brain Sciences MagdeburgMagdeburg, Germany

**Keywords:** DRD2, TaqIA, dopamine, genetic, motivation, interference processing, flanker, fMRI

## Abstract

Dopamine has been implicated in the fine-tuning of complex cognitive and motor function and also in the anticipation of future rewards. This dual function of dopamine suggests that dopamine might be involved in the generation of active motivated behavior. The DRD2 TaqIA polymorphism of the dopamine D2 receptor gene (rs1800497) has previously been suggested to affect striatal function with carriers of the less common A1 allele exhibiting reduced striatal D2 receptor density and increased risk for addiction. Here we aimed to investigate the influences of DRD2 TaqIA genotype on the modulation of interference processing by reward and punishment. Forty-six young, healthy volunteers participated in a behavioral experiment, and 32 underwent functional magnetic resonance imaging (fMRI). Participants performed a flanker task with a motivation manipulation (monetary reward, monetary loss, neither, or both). Reaction times (RTs) were shorter in motivated flanker trials, irrespective of congruency. In the fMRI experiment motivation was associated with reduced prefrontal activation during incongruent vs. congruent flanker trials, possibly reflecting increased processing efficiency. DRD2 TaqIA genotype did not affect overall RTs, but interacted with motivation on the congruency-related RT differences, with A1 carriers showing smaller interference effects to reward alone and A2 homozygotes exhibiting a specific interference reduction during combined reward (REW) and punishment trials (PUN). In fMRI, anterior cingulate activity showed a similar pattern of genotype-related modulation. Additionally, A1 carriers showed increased anterior insula activation relative to A2 homozygotes. Our results point to a role for genetic variations of the dopaminergic system in individual differences of cognition-motivation interaction.

## Introduction

The ability to adapt oneself to uncertain, changeable needs of the environment is considered as an outstanding human skill (Collins and Koechlin, 2012). These competences comprise the decision making based on exploration, adaptation to found conditions, anticipation of expected results or risks of a given action and a suitable choice from a variety of possible responses to a stimulus (Royall et al., 2002; Gilbert and Burgess, 2008; Collins and Koechlin, 2012). This complex set of skills is often subsumed under the term *Executive Functions* (EF), a somewhat diffuse umbrella term that attempts to capture the heterogeneity of the psychological processes involved.

Despite their apparent heterogeneity, the brain processes typically considered as EF can be subdivided into three core functions: inhibition (including the control of interference), working memory, and cognitive flexibility (Miyake et al., 2000; Diamond, 2013). These core functions support more complex cognitive functions like planning or problem solving and thus have a broad impact on human behavior and social interactions, affecting quality of life, job success as well as physical and mental health (Diamond, 2013).

Behavioral and neural manifestations of EF can be investigated in an experimental setting using a variety of well-established paradigms. For example, inhibitory processes can be investigated with the flanker task (Eriksen and Eriksen, 1974), the Simon task (Simon and Berbaum, 1990) or the Stroop task (MacLeod, 1991). In addition to inhibitory processes, successful performance of the flanker task also depends upon selective attention (Posner and Petersen, 1990; Diamond, 2013). Numerous variations of the flanker task exist, but their common feature is that participants are required to focus on a centrally presented target stimulus while ignoring flanking distractor stimuli. The performance of incongruent trials, during which the target stimulus and the flanking stimuli activate different possible reactions (i.e., responding to the central arrow in >>><>>>), is typically contrasted to the performance of congruent trials, during which the target and the distractors jointly activate one single choice of action (i.e., responding to the central arrow in >>>>>>>). Behaviorally, such interference in flanker tasks is characterized by concomitantly occurring slower reaction times (RTs) and higher error rates in incongruent as compared to congruent trials (Botvinick et al., 1999; Casey et al., 2000; Botvinick et al., 2004; Richter et al., 2011; Bugg and Crump, 2012). Further research suggests that the flanker task does not only allow the investigation of inhibition performance, but also action monitoring and error detection (Ullsperger and Von Cramon, 2004).

At the level of neural circuits, the prefrontal cortex (PFC) is widely considered to be the key neuroanatomical structure mediating EF. The intrinsic organization of the frontal lobes is complex, and a growing body of clinical studies provides evidence for heterogeneous effects of lesions in distinct PFC subregions on different subprocesses of executive functioning (Funahashi, 2001; Royall et al., 2002; Elliott, 2003). While most neuroimaging research on EF has focused on frontal brain structures like the anterior cingulate cortex (ACC) and the lateral PFC, these structures typically co-activate with parietal cortical regions (Roberts and Hall, 2008), reflecting large-scale attention networks that also show increased connectivity during rest (Fox et al., 2006). Moreover, the PFC interacts with subcortical structures, most notably the striatum and the thalamus (Casey et al., 2000; Fan et al., 2005; Posner and Rothbart, 2007).

Because EF, at least to a large extent, mediate goal-directed behavior, it is conceivable that stimuli associated with potential positive or negative reinforcers are likely to undergo preferential processing (Adcock et al., 2006; Boksem et al., 2008; Krebs et al., 2009; Richter et al., 2013), but, when task-irrelevant, also interfere with the task at hand and influence its neural underpinnings (Wiswede et al., 2009; Padmala and Pessoa, 2010; Richter et al., 2011). The association of a stimulus with the possibility to obtain a reward or to avoid an aversive outcome typically renders this stimulus highly salient (Boksem et al., 2008). To elucidate how processes of inhibition and error detection are modulated by such salience, the flanker task can be modified by introducing trials in which participants can receive rewards or avoid penalties upon correct performance (Boksem et al., 2008; Engelmann et al., 2009; Hubner and Schlosser, 2010). Boksem and colleagues investigated the relationship between punishment/reward sensitivity (assessed with the Behavioral Inhibition System and Behavioral Activation System questionnaires, BIS/BAS) and electrophysiological correlates of error processing in the flanker task, demonstrating that individual differences in reward and punishment sensitivity affected the amplitude of error-related event-related potential (ERP) components in flanker trials that were associated with reward or punishment, respectively (Boksem et al., 2008).

Additional evidence for a modulation of PFC/dACC-dependent inhibitory control by motivation comes from stop signal and Stroop tasks. Padmala and Pessoa (2010) used the stop signal-paradigm to investigate the neural mechanisms of cognition-motivation interactions during response inhibition. Selective rewarding of correct go-reactions was associated with longer inhibitory RTs in the rewarded relative to the control condition and with reduced PFC activation in rewarded trials. Compatibly, Krebs et al. (2010) observed that reward anticipation exerted beneficial behavioral effects on Stroop task performance, but reward-associated stimuli also impaired the processing of neutral stimuli.

Converging evidence from patient studies, psychopharmacology and genetic investigations suggests that variability of prefrontal dopaminergic neurotransmission contributes substantially to the widely observed individual differences in PFC-dependent EF (Mattay et al., 2003; Meyer-Lindenberg and Weinberger, 2006; Stelzel et al., 2010; Barnes et al., 2011; Tan et al., 2012). Most studies investigating the impact of the dopaminergic system on PFC function have focused on catechol-O-methyl transferase (COMT), an enzyme primarily involved in cortical, but not striatal dopamine clearance (Tunbridge et al., 2006), but there is increasing evidence for a delicately balanced mutual regulation of prefrontal and striatal dopamine turnover (Meyer-Lindenberg et al., 2002, 2005, 2007). The dopamine receptor D2 (DRD2) is the predominant postsynaptic dopamine receptor in the striatum, but sparsely expressed in the PFC. Presynaptic, autoinhibitory D2 receptors, on the other hand, play an important role in the regulation of dopamine release throughout the brain. Given this dual role of DRD2, it seems plausible that genetically mediated individual differences of DRD2 expression affect both human striatal and prefrontal neural processes. A commonly investigated single nucleotide polymorphism (SNP) linked to the DRD2 gene on chromosome 11q22-23 is the so-called TaqIA polymorphism, which is characterized by a polymorphic restriction site. The TaqIA polymorphism has been repeatedly associated with alterations of striatal dopaminergic neurotransmission. Despite the fact that the underlying molecular mechanisms are yet not fully understood, a number of studies have provided converging evidence for reduced DRD2 expression in homozygous and heterozygous carriers of the less common A1 allele relative to homozygotes of the A2 allele. *Post mortem* investigations and positron emission tomography (PET) suggest that A1 carriers show a 30–40% decrease in DRD2 density compared to A2 homozygotes in the striatum (Thompson et al., 1997; Pohjalainen et al., 1998; Jonsson et al., 1999; Ritchie and Noble, 2003). One study employing single photon emission tomography (SPECT) did not find a difference in D2 receptor binding between A1 carriers and A2 homozygotes (Laruelle et al., 1998), but that study was later criticized for the combination of healthy participants and patients with schizophrenia in a sample and for the low resolution of the SPECT method (Ritchie and Noble, 2003). Moreover, A1 carriers have been reported to exhibit increased striatal dopamine synthesis (Laakso et al., 2005), possibly reflecting reduced autoinhibitory signaling from presynaptic D2 receptors. In healthy human volunteers, DRD2 TaqIA has been shown to affect neural mechanisms of reward processing, compatible with the high levels of DRD2 expression in the striatum (Lee et al., 2007), and similar effects have been observed for other genetic variations that affect D2 receptor availability (Pecina et al., 2013). In light of the above-mentioned structural and functional connectivity between the PFC and the striatum and the regulation of dopamine release via autoinhibitory presynaptic D2 receptors, it seems plausible that DRD2 TaqIA also modulates PFC-dependent EFs. Indeed, DRD2 TaqIA has been demonstrated to affect task switching and working memory-related processes, the latter in epistatic interaction with COMT Val108/158Met genotype (Stelzel et al., 2009, 2010; Garcia-Garcia et al., 2011).

The reported influences of DRD2 TaqIA on individual differences in prefrontal and striatal function are likely to be particularly pronounced when cognitive processes depend directly on fronto-striatal interactions. In line with this notion, motivation-based probabilistic learning or reversal learning have been shown to be affected by the polymorphism at the levels of both behavior and neural correlates, with A1 carriers being less successful in predicting negative outcomes and showing diminished recruitment of PFC and striatum during negative feedback processing and reversal learning (Klein et al., 2007; Jocham et al., 2009).

The tasks employed by Klein, Jocham and colleagues depend upon the direct interaction of the PFC and the striatum. Here we aimed to investigate effects of DRD2 TaqIA genotype on the modulation of primarily PFC-dependent inhibitory control and action monitoring by motivational processes, i.e., the anticipation of monetary gain or loss. We employed a modified flanker task during which, in a subset of the trials, participants could receive a reward, or avoid a punishment, or both. Recent evidence from animal studies suggests that the combination of appetitive and aversive reinforcement is associated with more pronounced improvement of learning performance than either one type of reinforcement alone (Ilango et al., 2010). Aiming to generalize this observation to human EFs, we also included a combined reward and loss condition in the task. Participants were genotyped for the DRD2 TaqIA polymorphism and grouped into A1 carriers and non-carriers. In a first behavioral experiment, we hypothesized that behavioral responses to incongruent flanker trials would be faster, and possibly more accurate, in reward-associated or punishment-associated flanker trials, and that, in line with their increased risk for reward-related disorders like addiction (Noble, 2003; Wang et al., 2013), A1 carriers would show more pronounced motivation-related modulation of the flanker trials. At a neural level, we hypothesized that A1 carriers and non-carriers would exhibit differential activation patterns in brain regions associated with conflict processing like the dACC and structures associated with motivational processing, like the striatum and the insula.

## Materials and methods

### Participants

Participants were recruited from a cohort of 615 young (behavioral study: age range 18–30 years, mean 23.65 ± 2.86; fMRI study: age range 19–30 years, mean 23.00 ± 2.51), healthy volunteers of a large-scale behavioral genetic study conducted at the Leibniz-Institute for Neurobiology, Magdeburg. Based on the assumption that a possible small effect of genes may not only require a large number of volunteers but also a strict control of non-genetic factors (Lee et al., 2007), participants were assessed for several exclusion criteria. All participants were right-handed according to self-report, not genetically related, and had obtained at least a university entrance diploma (*Abitur*). Importantly, all participants had undergone routine clinical interview to exclude present or past neurological or psychiatric illness, alcohol, or drug abuse, use of centrally-acting medication, the presence of psychosis or bipolar disorder in a first-degree relative, and additionally, given the design of the experiment, frequent gambling. For both studies, the behavioral and the fMRI experiment, two participants were invited for piloting of the paradigm. Their data were not used for subsequent analyses. The final study sample consisted of 46 volunteers in the behavioral study and 32 participants in the fMRI study, with no overlap between the experiments. All participants gave written informed consent in accordance with the Declaration of Helsinki and received financial compensation for participation. The work was approved by the Ethics Committee of the University of Magdeburg, Faculty of Medicine.

### Genotyping

Genomic DNA was extracted from blood leukocytes using the GeneMole® automated system (Mole Genetics AS, Lysaker, Norway) according to the manufacturer's protocol. Genotyping was performed using PCR followed by allele-specific restriction analysis using previously described primers (Grandy et al., 1989). Briefly, the DNA fragment on Chr 11q23.1 containing the DRD2 TaqIA polymorphism (NCBI accession number: rs1800497) was amplified using the primers DRD2-F: 5′-CCGTCGACGGCTGGCCAAGTTGTCTA-3′ and DRD2-B: 5′-CCGTCGACCCTTCCTGAGTGTCATCA-3′ and standard Taq polymerase (Qiagen and Fermentas). PCR products were digested with *TaqI* (Fermentas), yielding two fragments (130 + 180 bp) for the A2 allele or a single fragment (310 bp) for the A1 allele. DNA fragments were separated on a 2.5% ethidium bromide-stained agarose gel and visualized under UV light. Because the COMT Val108/158Met polymorphism (NCBI accession number: rs4680) has previously been linked to individual differences in both PFC function and reward processing (Egan et al., 2001; Schmack et al., 2008; Wimber et al., 2011), participants were also genotyped for rs4680 using PCR and restriction with NlaIII (Schott et al., 2006; Wimber et al., 2011; details available upon request).

### Behavioral study

#### Paradigm

**Figure 1:**
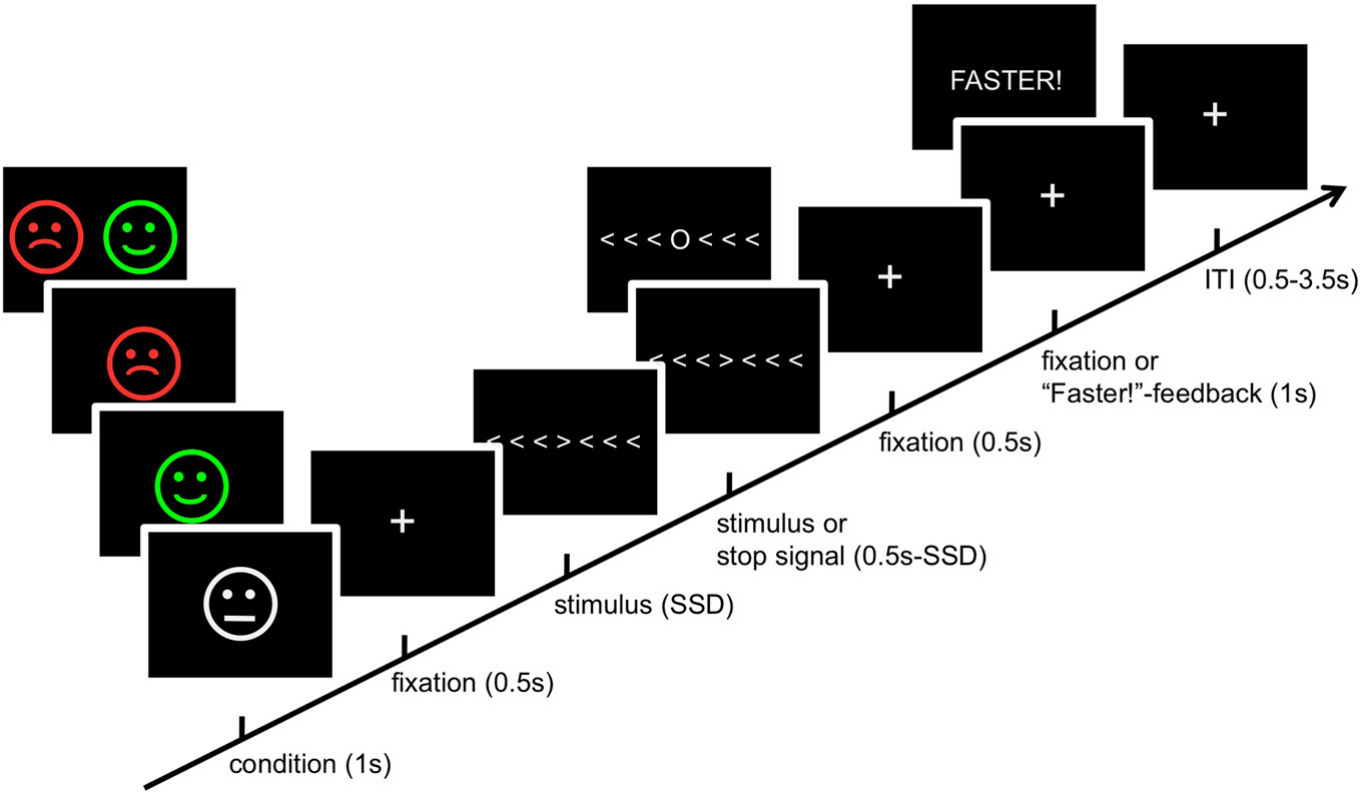
**Schematic illustration of the experimental paradigm**. See Materials and Methods section for details. SSD, stop-signal delay; ITI, inter trial interval.

We employed a modified Eriksen flanker task (Eriksen and Eriksen, 1974) with a motivation manipulation (Boksem et al., 2008). Participants were instructed to fixate a central target arrow and to indicate whether it was pointing to the left or to the right by pressing a button with the index or middle finger of the right hand. They had to ignore six distractor arrows with the same (congruent condition), the opposite (incongruent condition) or random (any three left and three right) orientation. Trials were grouped into four types of motivational categories. In reward trials (REW), volunteers were rewarded with 5 ct for fast and correct responses. Conversely, in punishment trials (PUN), they were punished for incorrect, slow or missing responses by the loss of 5 ct. These two conditions were complemented by neutral trials (NEU) in which responses were associated with neither gain nor loss and with trials in which fast and correct responses were rewarded and incorrect, slow or omitted responses were punished (combination trials—COM). Each condition constituted 25% of the trials, and participants were notified about the upcoming trial type before each trial by presentation of neutral, positive, or negative cartoon face (neutral faces, smilies and frownies; see Figure 1). RTs were monitored throughout the course of the experiment. RTs exceeding the current mean RT by more than one standard deviation (SD) were considered too slow, and participants received a feedback (“Faster!”) whenever it was exceeded. Accuracy feedback was not delivered.

As in a number of previous studies, the flanker task was combined with a stop-signal paradigm (Logan et al., 1984; Krämer et al., 2007; Boehler et al., 2009). Infrequently (on 20% of the trials), a circle instead of the target arrow was presented, signaling the participants to suppress their response. We used an adaptive short stop-signal delay (SSD) to yield an approximately equal number of signal-inhibit and signal-respond trials (Krämer et al., 2007; Boehler et al., 2009). The SSD was calculated online separate for each motivation condition. Participants were informed that rewards and punishments would never be delivered in stop trials, regardless of their inhibition performance. An example trial and the overview of the trial timing are displayed in Figure 1.

The experiment consisted of four runs with 144 trials per run (including 24 stop trials). Each run was counterbalanced for experimental conditions and direction of the target arrow. The currently earned amount of money was displayed after each run. Participants were tested alone or in groups of no more than three persons. Before the experiment they were instructed using a standardized written instruction, followed by the opportunity to ask questions. Before the actual experiment, participants performed a training phase consisting of 42 trials (12 stop trials) during which an accuracy feedback was delivered. In this training the starting value of the RT limit was calculated. Data of the training phase were not analyzed further. Participants could earn up to 9 Euros (mean = 5.57 Euros ± 1.53 Euros).

#### Statistical analysis

To examine the influence of the DRD2 TaqIA polymorphism on flanker performance and its modulation by reward and punishment, flanker trials were analyzed with respect to the percentage of incorrect responses and the RTs of correct responses. Correct and incorrect reactions between 200 and 1000 ms after stimulus onset were analyzed. As a measure of interference processing the difference between congruent and incongruent trials (congruency effect) was calculated. Analyses of variance (ANOVAs) for repeated measures were calculated for each dependent variable with the motivation condition as within-subject factor and DRD2 TaqIA genotype as between-subject factor. Degrees of freedom were corrected for non-sphericity using the Greenhouse-Geisser correction.

### Functional MRI experiment

#### Paradigm

The design of the task used in the behavioral study was simplified and adapted for the purposes of fMRI. As the random trials yielded accuracy rates and RTs that lay in between those of the congruent and the incongruent condition, we did not include random trials in the fMRI study, thereby increasing the number of congruent and incongruent trials contributing to the fMRI signal. Furthermore, the potential reward and punishment were increased from 5 to 20 ct, and participants received further 6 Euros to compensate for travel expenses, which they were told after the experiment. The trial timing of the events was the same as in the behavioral study, but the inter-trial interval was increased and jittered between 4 and 8 s, using a near-exponential jitter to optimize the estimation of the trial-specific BOLD responses (Hinrichs et al., 2000). In total, there were four runs with 96 trials each (16 stop trials). The training phase (36 trials including 6 stop trials) was performed outside the MR tomograph. Participants could earn up to 32 Euros (24.42 ± 3.48 Euros; plus 6 Euros).

#### Image acquisition

Four runs of 390 T2^*^-weighted echo-planar images (EPIs) per run were acquired on a GE Signa 1.5 T magnetic resonance system (General Electric Medical Systems) in an interleaved acquisition order (23 axial slices, odds first; voxel size = 3.13 mm × 3.13 mm × 4 mm + 1 mm gap; *TR* = 2 s; *TE* = 35 ms). Six EPIs were acquired before each run to allow for magnetic field stabilization and discarded from data analysis. Because proton-density (PD)-weighted MR images possess a good contrast for gray vs. white matter in the striatum and midbrain (D'Ardenne et al., 2008; Schott et al., 2008), a co-planar PD-weighted MR image was acquired and used for improved spatial normalization.

#### Data processing and analysis

Data analysis was carried out using Statistical Parametric Mapping (SPM8, Wellcome Department of Imaging Neuroscience, Institute of Neurology, London, UK). EPIs were corrected for acquisition delay and head motion. The co-planar PD-weighted image was coregistered to the mean image obtained from motion correction and used to determine normalization parameters for spatial normalization to the Montreal Neurological Institute (MNI) stereotactic coordinate system (voxel size = 3 × 3 × 3 mm). Data were smoothed using a Gaussian kernel of 8 × 8 × 8 mm, and a high-pass filter with a cut-off of 128 s was applied to the data.

Statistical analysis was performed using a two-stage mixed effects model. At the first stage, the hemodynamic response was modeled by convolving a delta function at stimulus onset with a canonical HRF (Friston et al., 1998). The resulting time courses were downsampled to the temporal resolution of fMRI scanning (1/*TR* = 0.5 Hz) to form covariates of a general linear model (GLM). The model included separate covariates for each condition of interest (correct responses in the conditions NEU-congruent, NEU-incongruent, REW-congruent, REW-incongruent, PUN-congruent, PUN-incongruent, COM-congruent, and COM-incongruent). The model included also covariates of no interest, namely incorrect responses, a feedback regressor, four stop-trial regressors for each motivation condition, the instruction screen, and the six rigid-body movement parameters determined from motion correction, plus a single constant representing the mean over scans. Model estimation was performed using a restricted maximum likelihood fit.

**Figure A1:**
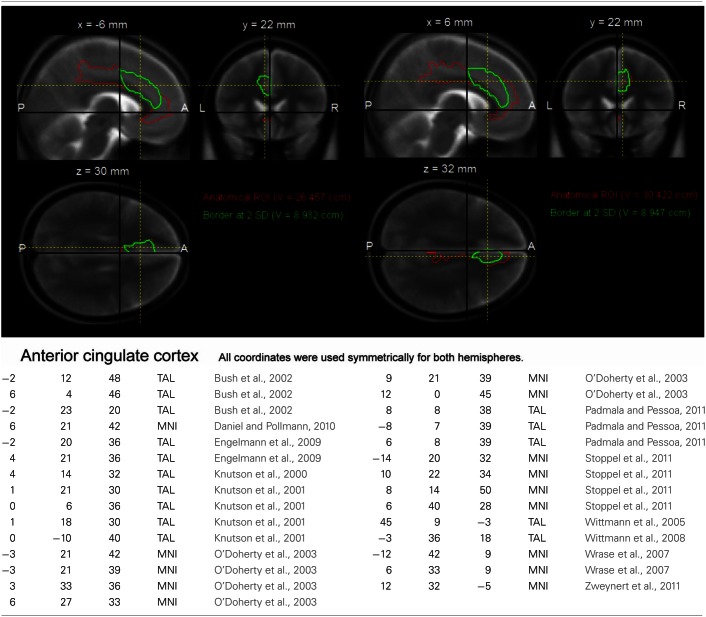
**Literature-based ROI of the dorsal ACC**.

**Figure A2:**
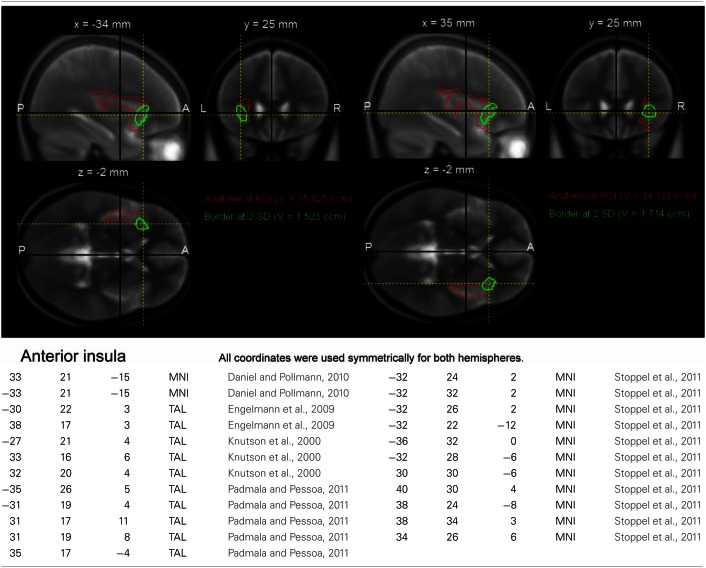
**Literature-based ROI of the anterior insula**.

**Figure A3:**
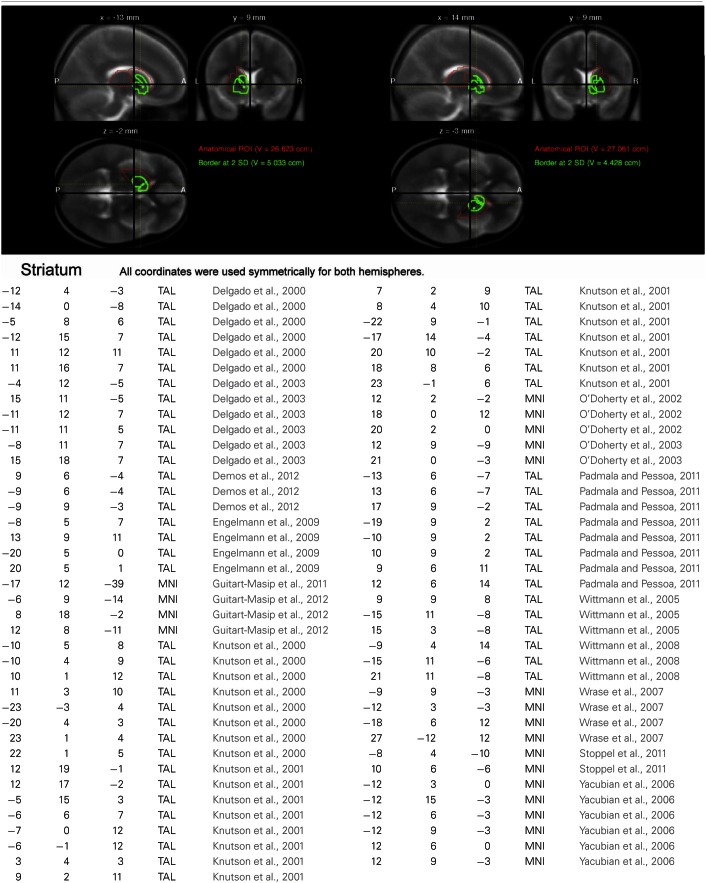
**Literature-based ROI of the striatum**.

At the second stage of the model, the conditions of interest separated by genotype were submitted to second level random effect analyses. Specifically, the within-subject factors congruency and motivation were submitted to a full-factorial ANOVA, with genotype as between-subject factor. Because of our strong *a priori* hypotheses regarding brain regions previously implicated in interference processing and motivation, several regions of interest (ROIs) were defined. The ROI of the dorsolateral prefrontal cortex (DLPFC) was generated using the automated anatomical labeling (AAL) of the superior and middle frontal gyrus implemented in the WFU Pickatlas (Wake Forest University), and ROIs of the ACC, the anterior insula and striatum were generated using a previously described literature-based probabilistic approach (Schubert et al., 2008; Zweynert et al., 2011; see Figures A1–A3). The *a priori* statistical threshold was set to *p* = 0.05 family wise error (FWE)-corrected for all comparisons, with the correction applied to ROI volumes for regions with *a priori* hypotheses, and an additional Bonferroni correction was applied to correct for the number of ROIs (*n* = 8). Coordinates are given in MNI space. To further verify reliability of genetically driven between-group differences and reduce the influence of outliers, confidence intervals were estimated for the local maxima using bootstrap resampling and the percentile-t method (Schott et al., 2006; Wimber et al., 2011). For visualization purposes, activations were superimposed onto the MNI template image provided by MRIcron (http://www.mccauslandcenter.sc.edu/mricro/mricron/).

## Results

### Genotyping

Among the 615 participants in the original cohort who were genotyped for the DRD2 TaqIA polymorphism, we identified 22 A1 homozygotes, 210 heterozygotes, and 383 A2 homozygotes. The distribution was at Hardy-Weinberg equilibrium [χ^2^ = 1.08, *p* = 0.298]. Regarding the COMT Val108/158Met polymorphism, the sample included 164 Met homozygotes, 322 heterozygotes, and 129 Val homozygotes, and HWE was not violated [χ^2^ = 1.57, *p* = 0.210].

### Behavioral results

In the behavioral study the data of 46 young, healthy participants were analyzed (27 women, 19 men). The cohort consisted of one A1 homozygote, 23 heterozygotes and 22 A2 homozygotes. Thirty-two participants took part in the fMRI study (19 women, 13 men), including 15 heterozygote A1 carriers and 17 A2 homozygotes. Given the low number of A1 homozygous subjects (*n* = 1), A1 carriers (A1+: A1/A1 and A1/A2) were grouped together for all subsequent analyses. The groups A1+ and A1− (A2/A2) did not differ in gender distribution, mean age or in percentage of smokers. Because the COMT Val108/158Met polymorphism (rs4680) has previously been demonstrated to affect PFC function and reward processing, participants were also genotyped for this SNP, and the distribution of Val and Met alleles did not differ significantly between groups. For detailed demographic information see Table 1. Error rates and RTs across the different conditions are displayed in Table 2, separated by DRD2 TaqIA genotype.

**Table 1:** **Demographic data**.

	**A1+**	**A1−**	
**BEHAVIORAL EXPERIMENT**			
Women/Men	14/10	13/9	χ^2^ < 0.01; *p* = 0.958
Mean age	24.2 ± 2.9	23.1 ± 2.7	*t*_(44)_ = 1.28; *p* = 0.206
Smokers/Nonsmokers	8/16	6/16	χ^2^ = 0.20; *p* = 0.655
COMT mm/vm/vv	10/9/5	6/12/4	χ^2^ = 1.46; *p* = 0.483
**fMRI EXPERIMENT**			
Women/Men	10/5	9/8	χ^2^ = 0.62; *p* = 0.430
Mean age	22.3 ± 1.9	23.6 ± 2.9	*t*_(30)_ = −1.43; *p* = 0.162
Smokers/Nonsmokers	3/12	6/11	χ^2^ = 0.92; *p* = 0.337
COMT mm/vm/vv	3/8/4	5/7/5	χ^2^ = 0.56; *p* = 0.758

Gender distribution, age (average ± SD), number of smokers and nonsmokers and COMT Val108/158Met occurrence (mm, met homozygotes; vm, val/met heterozygotes; mm, met homozygotes) of the participants.

**Table 2:** **Descriptive statistics of the behavioral data**.

	**A1+**		**A1−**	
		**RT [ms]**	**Error rate [%]**	**RT [ms]**	**Error rate [%]**
**BEHAVIORAL EXPERIMENT**					
All trials		410 ± 48	6.8 ± 5.4	419 ± 55	7.1 ± 5.3
Congruent trials	NEU	378 ± 44	0.5 ± 1.3	389 ± 50	1.0 ± 2.0
	REW	368 ± 45	0.6 ± 1.6	372 ± 40	0.6 ± 1.7
	PUN	371 ± 41	0.7 ± 1.7	375 ± 40	0.6 ± 1.5
	COM	372 ± 40	0.0 ± 0.0	380 ± 41	0.4 ± 1.3
Incongruent trials	NEU	469 ± 49	13.7 ± 12.0	473 ± 79	14.4 ± 11.9
	REW	450 ± 48	16.8 ± 15.0	459 ± 61	13.9 ± 12.5
	PUN	455 ± 50	16.6 ± 14.6	468 ± 63	16.0 ± 12.7
	COM	458 ± 51	15.2 ± 12.1	458 ± 64	13.4 ± 10.3
Random trials	NEU	422 ± 51	3.4 ± 4.2	433 ± 63	6.9 ± 7.3
	REW	406 ± 52	5.7 ± 6.3	414 ± 62	6.4 ± 5.7
	PUN	408 ± 53	4.9 ± 5.3	425 ± 64	4.8 ± 5.8
	COM	409 ± 59	3.4 ± 3.8	423 ± 59	6.9 ± 6.4
**fMRI EXPERIMENT**					
All trials		438 ± 38	3.1 ± 3.2	440 ± 43	2.2 ± 2.5
Congruent trials	NEU	409 ± 34	0.3 ± 0.7	411 ± 43	0.2 ± 0.7
	REW	398 ± 40	1.1 ± 1.7	401 ± 42	0.2 ± 0.7
	PUN	399 ± 40	0.8 ± 1.7	403 ± 47	0.2 ± 1.0
	COM	400 ± 42	0.1 ± 0.5	403 ± 45	0.6 ± 1.6
Incongruent trials	NEU	484 ± 41	4.3 ± 3.6	484 ± 48	3.6 ± 4.1
	REW	475 ± 41	6.0 ± 7.8	474 ± 44	4.4 ± 4.5
	PUN	473 ± 40	7.1 ± 9.3	476 ± 45	4.7 ± 6.4
	COM	478 ± 40	4.7 ± 4.6	473 ± 45	3.4 ± 5.0

Mean reaction times (RT) of correct responses and error rates ± standard deviations (SD) are shown. NEU, condition with no reward or punishment; REW, rewarded condition; PUN, punished condition; COM, condition with reward and punishment.

#### Effects of congruency and motivation

Overall, participants responded fast and accurately. To test for genotype-related and task-related differences in behavioral performance, we computed ANOVAs for repeated measures with congruency and motivation as within-subject factors and genotype as between-subject factor. Replicating previous results (Botvinick et al., 1999; Casey et al., 2000; Botvinick et al., 2004; Richter et al., 2011; Bugg and Crump, 2012), we observed a main effect of flanker condition with higher error rates and slower RTs in the incongruent as compared to the congruent condition in both the behavioral [main effect of congruency: RT: *F*_(2, 88)_ = 360.26, *p* < 0.001; error rate: *F*_(2, 88)_ = 66.00; *p* < 0.001] and the fMRI experiment [RT: *F*_(1, 30)_ = 387.10, *p* < 0.001; error rate: *F*_(1, 30)_ = 26.73, *p* < 0.001]. In the behavioral study, error rates and RTs in the random trials lay in between those of the congruent and the incongruent trials, suggesting that the congruency effect depended on the number of distractors (Table 2). Motivational salience (i.e., the presence of reward, punishment, or both) was associated with shorter RTs in all motivated trials compared to the NEU in both the behavioral [main effect of motivation: *F*_(3, 132)_ = 36.18, *p* < 0.001] and the fMRI experiment [*F*_(3, 90)_ = 11.00, *p* < 0.001], while the error rates did not differ significantly across the different motivation conditions (all *p* > 0.074). In the behavioral study, the REW condition elicited the shortest RTs [REW vs. PUN: *t*_(45)_ = −3.98, *p* < 0.001; REW vs. COM: *t*_(45)_ = −4.12, *p* < 0.001; PUN vs. COM: *t*_(45)_ = −0.07, *p* = 0.947].

#### Genotype-related modulation of cognition-motivation interaction

Across flanker and motivation conditions there was no genotype-related difference in overall RTs [*Behavioral experiment:* A1 carriers: 410 ± 48 ms, A2/A2: 419 ± 55 ms; *t*_(44)_ = −0.58, *p* = 0.567; *fMRI experiment:* A1 carriers: 438 ± 38 ms, A2/A2: 440 ± 43 ms; *t*_(30)_ = −0.12, *p* = 0.905], suggesting that there were no genotype-related differences in sensorimotor function.

**Figure 2:**
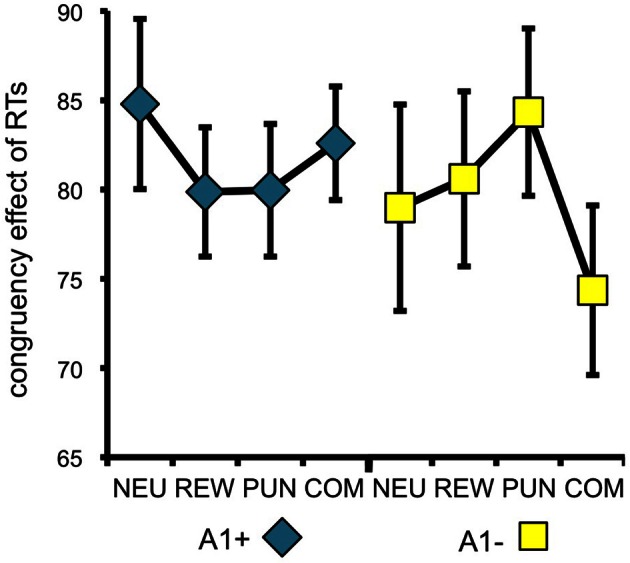
**Behavioral congruency effect**. Plots depict the difference between incongruent and congruent RTs for each motivation condition (± standard errors). Data from both experiments (behavioral and fMRI) are combined. Higher values indicate stronger distractor interference. The observed pattern suggests that A1 carries showed a small to moderate reduction of the RT difference (incongruent vs. congruent) in all motivated trials, particularly in the reward condition, whereas the RT difference reduction in A2 carriers was largely restricted to the combined condition [genotype by motivation interaction: *F*_(3, 228)_ = 2.96; *p* = 0.039]. NEU, neutral condition; REW, reward condition; PUN, punishment condition; COM, combined reward and punishment condition.

To specifically test for effects of genotype on interference processing and its modulation by motivational salience, we computed the behavioral congruency effects, i.e., the differences of error rates and RTs between incongruent and congruent trials, separated by motivation conditions. These values were the dependent variables in ANOVAs for repeated measures with motivation condition (NEU vs. REW vs. PUN vs. COM) as within-subject factor with four levels, and DRD2 TaqIA genotype (A1+ vs. A2/A2) as between-subject factor with two levels. The analysis of error rates revealed no significant effects of either factors motivation or genotype (all *p* > 0.120), but in the analysis of congruency-related RT differences, a significant motivation by genotype interaction was observed in the behavioral experiment [*F*_(3, 132)_ = 3.07, *p* = 0.039]. While this interaction effect was not significant in the (smaller) cohort of the fMRI experiment, it remained significant when combining the data of both experiments [*F*_(3, 225)_ = 2.96, *p* = 0.039; because of the differences in experimental design, the experiment—behavioral vs. fMRI—was included as a covariate of no interest in this ANOVA]. To explore the pattern underlying this interaction, we computed *post-hoc* paired *T*-tests on the RT congruency effects in the different motivation conditions, separated by DRD2 TaqIA genotype. Results of the *post-hoc* comparisons are displayed in Table 3 (both studies combined) and in Table A1 (both studies separately; note that *post-hoc* comparisons from the fMRI experiment are for illustrative purpose only, given the lack of an interaction effect in the ANOVA). In summary, the results of the *post-hoc* tests, albeit exploratory, suggest that A1 homozygotes showed a reduced congruency effect primarily in the rewarded condition (significant in the behavioral study only, see Table A1) and nominally benefitted from all motivated conditions, whereas A2 homozygotes showed smaller congruency-related RT differences in the combined condition relative to the conditions with reward or punishment alone (Figure 2, Tables 3, A1).

**Table 3:** **Behavioral data (*t*-statistics)**.

**Condition**	**A1+**		**A1−**	
	***t*_*38*_**	***p***	***t*_*38*_**	***p***
REW vs. NEU	−1.24	0.111	0.45	0.327
PUN vs. NEU	−1.35	0.093	1.40	0.085
COM vs. NEU	−0.57	0.288	−1.23	0.113
REW vs. PUN	−0.03	0.977	−1.10	0.279
COM vs. REW	0.94	0.178	−2.04	0.025^*^
COM vs. PUN	0.92	0.183	−3.54	<0.001^*^

Results of post-hoc paired T-tests testing for effects of the motivation conditions on the congruency effect of reaction times, separated by genotype group and collapsed across experiments. All p-values are one-tailed, except for the REW vs. PUN contrast for which we had no directed hypothesis. NEU, neutral condition; REW, reward condition; PUN, punishment condition; COM, combined reward and punishment condition.

* p < 0.05.

### Functional MRI results

All comparisons were based on a full-factorial ANOVA model with congruency (congruent vs. incongruent), motivation (NEU vs. REW vs. PUN vs. COM), and genotype (A1+ vs. A2/A2) as factors. An overview of the relevant comparisons in the regions of interest (dACC, DLPFC, insula, striatum) is displayed in Table 4.

**Table 4:** **Peak activation foci in the ROI analyses**.

	**Cluster size**	**Hemisphere**	***z*-value**	***x***	***y***	***z***
**EFFECTS OF CONGRUENCY AND MOTIVATION**						
**INCONGRUENT vs. CONGRUENT**						
Anterior insula	57	R	7.21^*^	33	23	1
	52	L	7.62^*^	−30	26	−2
Dorsolateral prefrontal cortex	66	R	5.73^*^	42	5	37
	48	L	5.38^*^	−30	−7	52
Anterior cingulate cortex	100	R	5.92^*^	9	14	46
	36	L	3.75	−6	5	46
Striatum	63	R	6.00^*^	12	5	−2
	22	L	4.04^*^	−9	8	4
**REW vs. NEU**						
Anterior insula	58	R	6.15^*^	33	23	−2
	36	L	4.37^*^	−30	29	−2
Dorsolateral prefrontal cortex	103	R	4.03	36	56	7
	64	R	3.93	30	5	52
	36	L	3.88	−21	−4	49
Anterior cingulate cortex	295	R	5.60^*^	6	32	28
	185	L/R	4.61^*^	0	32	28
Striatum	102	R	5.98^*^	9	17	−5
	93	L	6.64^*^	−9	14	−5
**PUN vs. NEU**						
Anterior insula	55	R	5.95^*^	33	23	1
	8	L	3.01	−36	20	1
Dorsolateral prefrontal cortex	48	L	4.56^*^	−33	−1	52
	14	L	3.98	−39	8	34
	40	L	3.94	−36	41	7
Anterior cingulate cortex	165	R	4.86^*^	6	26	31
Striatum	89	R	4.76^*^	12	11	−8
	100	L	5.04^*^	−9	11	−5
**COM vs. NEU**						
Anterior insula	56	R	6.39^*^	33	23	−2
	35	L	4.63^*^	−33	20	−11
Dorsolateral prefrontal cortex	96	R	5.45^*^	30	5	52
	167	R	5.02^*^	42	53	4
	49	L	4.66^*^	−33	−1	52
	24	L	4.03	−33	41	4
	12	L	3.97	−39	8	34
Anterior cingulate cortex	252	R	5.76^*^	6	32	28
	142	L/R	4.90^*^	0	35	31
Striatum	120	R	6.11^*^	12	17	−2
	153	L	6.36^*^	−12	17	−5
**REW vs. PUN**						
Anterior insula	4	L	3.38	−30	26	−5
Anterior cingulate cortex	52	R	3.75	6	41	16
	51	L/R	3.38	0	35	22
**PUN vs. REW**	−	−	−	−	−	−
**COM vs. REW**	−	−	−	−	−	−
**COM vs. PUN**						
Anterior insula	7	L	3.41	−33	20	−11
Anterior cingulate cortex	26	R	3.45	3	35	25
	45	L	4.12^*^	−3	38	25
**CONGRUENCY × MOTIVATION**						
Dorsolateral prefrontal cortex	37	L	4.05	−18	56	16
**GENOTYPE-RELATED EFFECTS**						
**A1+ vs. A1−**						
Anterior insula	15	L	4.08^*^	−30	20	−8
Dorsolateral prefrontal cortex	2	L	4.04	−27	−13	49
**A1− vs. A1+**	−	−	−	−	−	−
**CONGRUENCY × MOTIVATION × GENOTYPE**						
Anterior cingulate cortex	31	R	4.07^*^	9	38	28
	12	L	3.44	−3	38	31
Striatum	4	R	3.25	21	−1	−2

Clusters with peak activation p < 0.05, FWE-corrected for ROIs volume with their cluster extent at p < 0.005, uncorrected. Coordinates are given in MNI space (unit mm). R, right; L, left;

* p-values remained significant after Bonferroni correction for multiple ROIs (N = 8).

#### Effects of congruency and motivation

**Figure 3:**
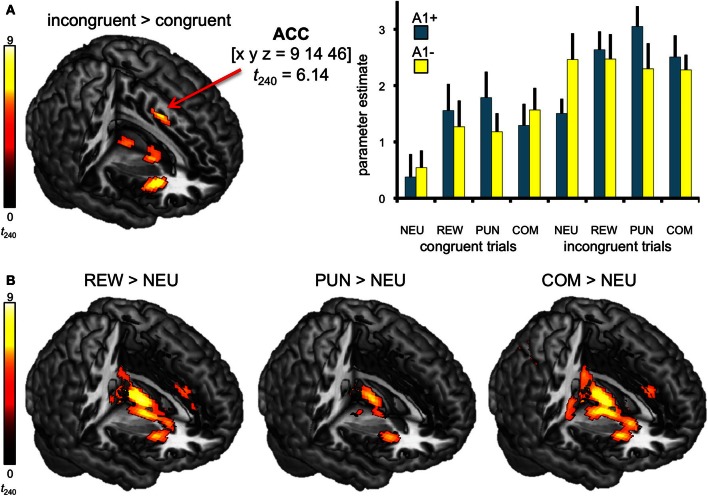
**Neural correlates of congruency and motivation. (A)** Effect of congruency. Incongruent trials elicited higher activity in dACC relative to congruent trials. Bar plots depict the corresponding parameter estimates of the parametric regressors at the ACC peak coordinate of the contrast incongruent vs. congruent trials are shown, separated by motivation conditions (± standard errors). **(B)** Neural correlates of motivational salience. Brain regions exhibiting motivation-related activation differences include the striatum, the anterior insula, and the ACC. All activation maps are superimposed on the MNI template brain provided by MRIcron. Contrasts were significant at *p* < 0.05, FWE-corrected. Coordinates are in MNI space. NEU, neutral condition; REW, reward condition; PUN, punishment condition; COM, combined reward and punishment condition.

In line with previous studies (Ridderinkhof et al., 2004; Ullsperger and Von Cramon, 2004), a one-tailed *T*-test comparing BOLD responses of incongruent and congruent trials revealed increased activity in distributed regions of the DLPFC and in the dorsal anterior cingulate cortex (dACC; see Figure 3A, Table 4).

The effect of motivational salience was tested by means of comparing the three motivated conditions to the neutral condition, using a one-tailed *T*-test. Irrespective of flanker condition and genotype, motivation-associated trials elicited higher BOLD responses in the bilateral striatum (Knutson et al., 2000; Wittmann et al., 2005) as well as in the ACC, the anterior insula, and in the bilateral lingual gyri when compared to neutral flanker (see Figure 3B, left panel). The anticipation of (avoidable) monetary punishment was associated with a similar pattern of brain activity increases, albeit of lower magnitude (Figure 3B, middle panel). Activations in the combined reward and punishment trials were largely comparable to those in the rewarded trials (Figure 3B, right panel).

**Figure 4:**
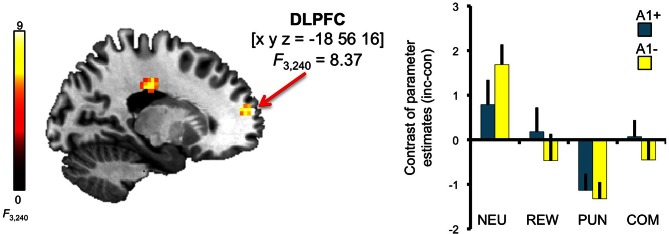
**Congruency by motivation interaction in the PFC**. A genotype-independent interaction of congruency and motivation was observed in the PFC (BA 10, bordering BA 46) where activation related to the incongruent versus congruent flanker condition was reduced in the motivated relative to neutral trials. This interaction effect was significant at *p* < 0.05, small-volume FWE-corrected for ROI volume. Activations are superimposed on the MNI template brain provided by MRIcron. Coordinates are in MNI space. Bar plots depict contrasts of parameter estimates (incongruent-congruent) at the peak coordinate separated by genotypes and motivation conditions. Error bars depict standard errors of the mean. NEU, neutral condition; REW, reward condition; PUN, punishment condition; COM, combined reward and punishment condition; INC, incongruent; CON, congruent.

A trend for a genotype-independent interaction of congruency and motivation was observed in our ROI of the DLPFC [*x*, *y*, *z* = −18, 56, 16; *F*_(3, 240)_ = 8.37; *p* = 0.036, FWE-corrected for ROI volume, but not significant after Bonferroni correction for multiple ROIs] where activation related to the incongruent flanker condition was reduced in the motivated trials relative to NEU (Figure 4). Further localization of the activation maximum using the BA map provided by MRIcron revealed that the cluster was located in the lateral portion of Brodmann area (BA) 10, bordering BA 46.

#### Genotype-related modulation of cognition-motivation interaction

**Figure 5:**
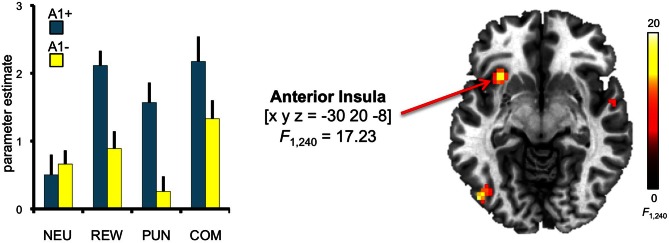
**Genotype-dependent modulation of insula activity**. A1 carriers exhibited increased activation of the anterior insula, when compared to A1- in the conditions with potential reward and punishment. This main effect of genotype was significant at *p* < 0.05, small-volume FWE-corrected for ROI volume. Activations are superimposed on the MNI template brain provided by MRIcron. Coordinates are in MNI space. Bar plots depict parameter estimates at the peak coordinate separated by genotypes and motivation conditions. Error bars depict standard errors of the mean. NEU, neutral condition; REW, reward condition; PUN, punishment condition; COM, combined reward and punishment condition.

To investigate potential effects of DRD2 TaqIA genotype on the motivational modulation of interference processing, we first computed the *F*-test comparison for the main effect of genotype. Compared to A2 homozygotes, A1 carriers exhibited increased activation of the left anterior insula [main effect of genotype: *x*, *y*, *z* = −30, 20, −8; *F*_(1, 240)_ = 17.23; *p* = 0.002, FWE-corrected for ROI volume] (Figure 5). To verify the reliability of the between-group differences, confidence intervals were estimated for the two genotype groups using bootstrap resampling and the percentile-t method (Schott et al., 2006). Between-group differences were reliable as indicated by non-overlapping 95 per cent confidence intervals in three motivated conditions (congruent REW, incongruent PUN, incongruent COM), but the confidence intervals in the neutral conditions were largely overlapping between genotype groups, raising the possibility that the genotype-related differences might be largely driven by the motivated conditions. To further explore this possibility, we performed an exploratory *post-hoc* masking analysis in which the main effect of genotype was inclusively masked with the genotype by motivation interaction contrast (thresholded at *p* < 0.05, uncorrected). The genotype-related activation difference in the left insula remained significant at *p* < 0.05, corrected for ROI volume, in this masking analysis.

**Figure 6:**
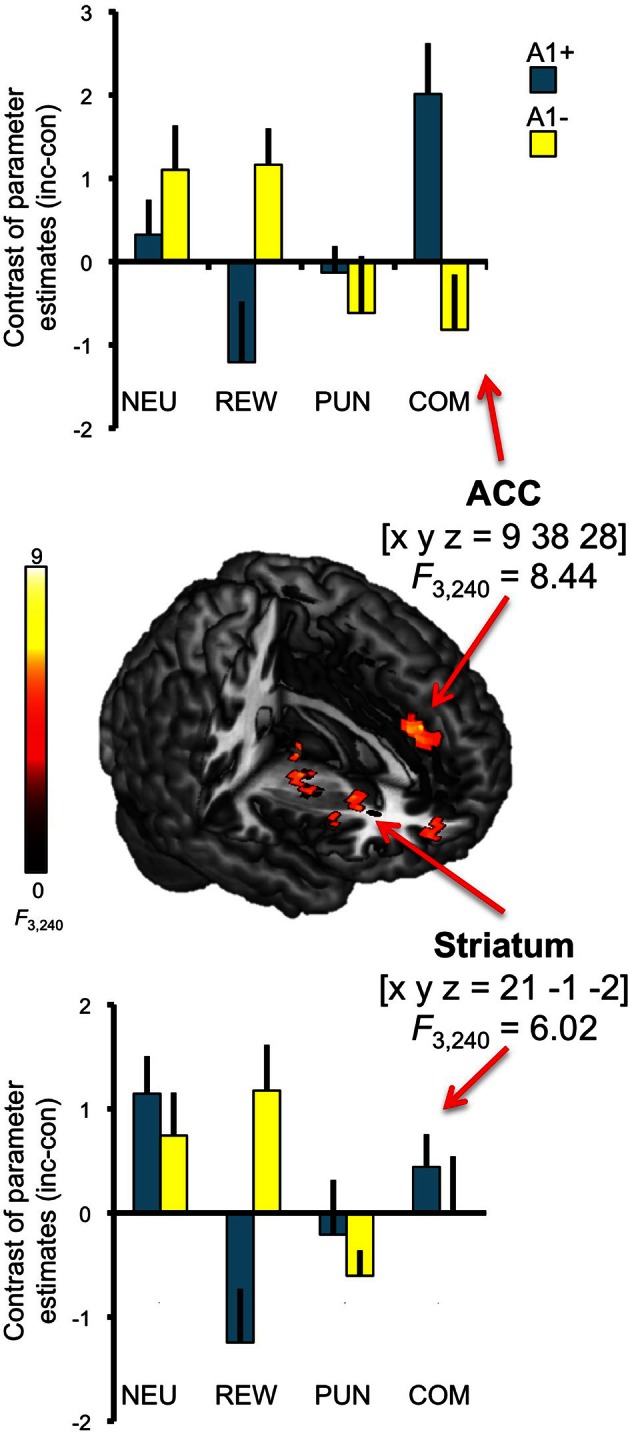
**Interaction of genotype, congruency, and motivation**. Complex genotype-dependent modulation of interference processing was observed in anterior cingulate cortex (ACC) and striatum. The three-way interaction of congruency × motivation × genotype is displayed, which was significant for the ACC at *p* < 0.05, small-volume FWE-corrected for ROI volumes. Activations are superimposed on the MNI template brain provided by MRIcron. Coordinates are in MNI space. Bar plots depict contrasts of parameter estimates at the peak coordinate separated by genotypes and motivation conditions. NEU, neutral condition; REW, reward condition; PUN, punishment condition; COM, combined reward and punishment condition; INC, incongruent; CON, congruent.

In addition to the main effect of genotype in the anterior insula, we observed a three-way interaction (congruency × motivation × genotype) in the ACC [*x*, *y*, *z* = 9, 38, 28; *F*_(3, 240)_ = 8.44; *p* = 0.006, FWE-corrected for ROI volume; see Figure 6, top]. *Post-hoc* two-sample *T*-tests over the contrasts of parameter estimates (incongruent vs. congruent) at the peak voxel in the right ACC revealed that A2 homozygotes showed higher activation in the trials with potential reward when compared to A1 carriers [ACC: *t*_(30)_ = −2.87; *p* = 0.007] while A1 carriers as compared to A2 homozygotes exhibited increased activation of the right ACC in the combined reward and punishment condition [*t*_(30)_ = 3.12; *p* = 0.004]. We also observed a trend for a three-way interaction in the right striatum [*x*, *y*, *z* = 21, −1, −2; *F*_(3, 240)_ = 6.02; *p* = 0.050, FWE-corrected for ROI volume; see Figure 6, bottom], but this did not survive Bonferroni correction for multiple ROIs.

#### Effects of the COMT Val108/158Met polymorphism on flanker-related brain activity

In an exploratory analysis regarding the effects of the well-characterized COMT Val108/158Met polymorphism on neural correlates of the flanker task, we observed a genotype by congruency interaction in the lateral PFC, but outside our *a priori* defined anatomical ROI of the DLPFC [*x*, *y*, *z* = 51, 17, 22; *F*_(2, 232)_ = 7.80; *p* < 0.001, uncorrected]. Specifically Val homozygotes showed relatively higher lateral PFC activation in incongruent trials relative to Met carriers. There were, however, no further interaction effects between COMT genotype and motivational salience.

## Discussion

In the present study, we investigated the influences of the DRD2 TaqIA polymorphism on the modulation of interference processing by reward and punishment. Motivational salience, i.e., the possibility to obtain a reward, to avoid a punishment, or both, was associated with shorter RTs in both the incongruent and congruent flanker condition. While the congruency-related RT difference did not differ between motivation conditions, functional MRI revealed a reduced congruency effect in the DLPFC during motivated trials, possibly reflecting increased processing efficiency. Moreover, we observed a complex interaction effect of motivation and genotype on the congruency-related RT differences in the behavioral experiment. This effect was not significant in the behavioral data of the fMRI experiment, but could still be observed when combining both datasets. Nominally, carriers of the less common DRD2 TaqIA A1 allele (A1+) with presumably lower D2 receptor density in striatum showed an, at least trendwise, improvement of interference processing in all motivated conditions (most strongly in the rewarded condition), whereas A2 carriers exhibited pronounced improvement during combined anticipation of reward or punishment as compared to either reward or punishment alone. At a neural level, genotype-related activation differences were observed in the anterior insula where A1 carriers showed increased task-related activation, and in the anterior cingulate, where a complex task by genotype interaction was observed.

### Effects of motivation on flanker performance and neural correlates

The motivation to obtain a reward or to avoid a loss was associated with shorter RTs in both, congruent and incongruent trials, while error rates did not show a significant modulation by motivational salience. Because of the dichotomous nature of accuracy rates and the considerable individual variability, the power to detect significant within- or between-group differences is limited, and RTs with their continuous distribution might be a more sensitive measure of motivation-related enhancement of cognitive processing, reflecting enhanced vigilance in motivated trials (Hardin et al., 2006). One might argue, however, that shorter RTs, when accompanied by reduced accuracy, might reflect impulsive responding rather than improved performance (Caldu et al., 2007). In the present study, error rates were nominally higher in the reward-related and punishment-related trials, but not in the combined condition. Given the overall low error rates and high variability, it is not possible to determine whether the RT decrease in motivated trials observed here might be to some degree related to impulsive responding. Reward anticipation has been demonstrated to promote responding, but to impair response inhibition in a probabilistic go/no-go task, but no such pattern has been observed for the anticipation of losses (Guitart-Masip et al., 2011). During interference processing in a Stroop task, on the other hand, accuracy was actually improved for rewarded trials (Krebs et al., 2010). Future studies employing more sensitive measures of accuracy are therefore needed to determine whether reward-related reductions of response times during performance of complex tasks reflects actual improvement of performance vs. a speed-accuracy tradeoff.

Despite the lack of a specific modulation of the RT congruency effect by motivation, at a neural level, we observed a congruency by motivation interaction in the PFC where motivational conditions were associated with reduced activation during processing of incongruent relative to congruent flanker trials. This prefrontal fMRI response reduction is well in line with previous studies suggesting that dopamine modulates processing efficiency in the PFC. Decreased PFC activation accompanied by comparable or even superior behavioral performance has previously been suggested to reflect higher processing efficiency, which has been reported in carriers of the (low-activity) COMT 158Met allele (Egan et al., 2001; Meyer-Lindenberg and Weinberger, 2006; Schott et al., 2006; Caldu et al., 2007) and in Parkinson's disease patients who received L-dopa (Mattay et al., 2002). Most studies reporting dopaminergic modulation of processing efficiency focused on the DLPFC. The activation cluster showing a congruency by motivation interaction in our study was located in the lateral portion of BA 10, in close proximity to BA 46. According to a common definition, BA 9 and 46 are referred to as DLPFC (Cieslik et al., 2012), but there is considerable heterogeneity in the literature regarding the precise delineation of the DLPFC, with several authors referring to at least parts of BA 8, 10, and 45 belonging to the DLPFC (Sarazin et al., 1998; Nitschke et al., 2006; Leung and Cai, 2007), while others have listed BA 46 as part of the ventrolateral PFC (Arango et al., 1995). *In vivo* segmentation of PFC subregions is also somewhat problematic, since most definitions are based on *post mortem* cytoarchitectonic mapping. The precise localization of the prefrontal cluster showing a congruency by motivation interaction to a subregion within the PFC remains thus somewhat speculative. It should be noted, though that its presumed position at the intersection of the DLPFC and the frontopolar cortex is in line with a previous study demonstrating joint deactivation of BA 10 during working memory and reward processing (Pochon et al., 2002) and with recent evidence for pronounced functional connectivity between the anterior portion of the DLPFC and the dACC (Cieslik et al., 2012).

Given the lack of a specific behavioral effect of motivation on the RT congruency effect, our results do not allow us to directly infer that the reduced overall RTs in motivated conditions are the result of increased prefrontal processing efficiency. On the other hand, more generally speaking, the co-occurrence of reduced RTs and decreased DLPFC activation to incongruent trials is at least indicative for a relationship between motivational processes, which are known to elicit dopamine release (Koepp et al., 1998; Schott et al., 2008) and PFC-dependent cognitive processing. In line with this notion, an exploratory analysis within the present study suggested that the COMT 158Val allele, which has been linked to lower prefrontal dopamine availability, was associated with increased lateral PFC activation to incongruent flanker trials.

Several previous studies have investigated the influence of reward and punishment on cognitive tasks, but little is thus far known about their combined effects. Recent animal studies on discrimination learning of frequency-modulated (FM) tones (Ilango et al., 2010) suggest that a combination of both reward and punishment might be associated with particularly strong performance enhancement. In a shuttle box paradigm, Mongolian gerbils were motivated by either appetitive reinforcement (brain stimulation reward) or by aversive reinforcement (avoidance of an electrical footshock), or by a combination of both. Compared to either reinforcement condition alone, the combination of both potentiated speed of acquisition and maximum performance while reducing later extinction. In the study by Ilango and colleagues, reward and punishment were qualitatively distinct (brain stimulation reward vs. foot shock), whereas in our study, the difference between the reward conditions was rather a quantitative one (monetary gain vs. loss). Therefore, the COM condition could to some extent be considered as a reward condition, although it would elicit larger prediction errors than the REW condition. On the other hand, the behavioral pattern observed here speaks against a merely quantitative difference. Namely, while the overall RT reduction across conditions was at least nominally less pronounced in the COM relative to the REW and PUN conditions, the COM condition was the one to show the strongest trend of a motivation-related reduction of the RT congruency effect (for a further interaction with DRD2 genotype, see below). One possible reason for this could be that participants might have slowed down their responses to some extent in the combination condition, in order to maximize accuracy. Indeed, accuracy was nominally higher in the COM condition as compared to the REW and PUN conditions, but these differences were not significant, possibly due to lack of statistical power given the overall high accuracy. Further experiments are needed to clarify whether the combination of both appetitive and aversive reinforcement indeed leads to a shift from speed to accuracy. As a potential limitation, it should also be noted that the size of the cue was larger in the combined condition (Figure 1), which could have distracted the participants from fixation of the target arrow after the cue (*Note:* Pilot data from a recent follow-up experiment with cues of equal size does not support the latter explanation).

### Genetic variability of D2 receptor availability interacts with motivational modulation of cognitive performance

DRD2 TaqIA genotype did not affect overall processing speed, but the congruency-related RT differences, suggesting that its effects cannot be explained by genotype-related differences in sensorimotor processing. Group-specific analysis of the congruency-related RT differences in each motivation condition revealed that A1 carriers exhibited improved interference processing in motivated, particularly rewarded trials (albeit significantly so only in the behavioral experiment), whereas the A2 homozygotes benefitted primarily from the combined reward and punishment condition. DRD2 TaqIA has been extensively investigated in neuropsychiatric disorders with presumed dopaminergic dysfunction, and the A1 allele has been associated with increased risk for disorders like substance abuse and pathological gambling or obesity, whereas the A2 allele has been implicated in the genetic risk for schizophrenia (Comings et al., 1996; Noble, 2003; Dubertret et al., 2004; Klein et al., 2007; Wang et al., 2013). Moreover, studies in healthy humans have suggested a role of the DRD2 TaqIA A1 variant in approach-related personality traits (Noble et al., 1998; Reuter et al., 2006; Lee et al., 2007; Smillie et al., 2010). While our finding that A1 carriers exhibit a reduction of the congruency-related RT difference in rewarded trials (and nominally in all motivated conditions) is compatible with the notion that A1 carriers might be more sensitive to rewards and losses, the observation that A2 carriers specifically benefitted from the combined condition was unexpected. The A2 allele has been linked to higher D2 receptor expression in the striatum (see, for example, Ritchie and Noble, 2003). Studies in transgenic mice have shown that even transient overexpression of D2 receptors in the striatum leads to persistent alterations of PFC-dependent cognitive functions, particularly working memory and cognitive flexibility (Kellendonk et al., 2006), and electrophysiological investigations further suggest that these alterations might be related to reduced inhibitory neurotransmission and lower prefrontal dopamine sensitivity (Li et al., 2011). Because levels of D2 receptor overexpression are higher than the described genotype-related D2 receptor expression differences in humans, inferences from these transgenic animal studies to effects of DRD2 TaqIA genotype effects must be considered tentative. If prefrontal dopamine sensitivity was reduced in A2 homozygotes, this might indeed provide a potential explanation for our behavioral results, namely, while reward or punishment alone might be insufficient to raise prefrontal dopamine availability to a level that allows improved interference processing, the combined condition might be associated with a further increase of prefrontal dopamine that might in turn enable a performance advantage in the A2 homozygotes. In A1 carriers, on the other hand, the congruency-related RT difference was at least nominally larger in the combined condition relative to either reward or punishment alone and not significantly different from the neutral condition. If the combined condition was indeed associated with higher prefrontal dopamine release than either reward or punishment alone, the resulting dopamine levels in A1 carriers might be too high for optimal performance, compatible with the model of an inverse U-shaped function of prefrontal dopamine (Meyer-Lindenberg and Weinberger, 2006).

At a neural level, a complex task by genotype interaction was observed in the dACC (Figure 6, top). Compared to A2 homozygotes, A1 carriers exhibited relatively reduced dACC activation to incongruent vs. congruent flanker trials in the REW condition, while this pattern reversed in the COM condition, meaning that both groups exhibited lower dACC activation in the condition in which they showed their most pronounced reduction of the congruency-related RT difference. In the DLPFC, lower activation accompanied by comparable or superior performance has been suggested to reflect higher processing efficiency (Meyer-Lindenberg and Weinberger, 2006; see also above), and at least one study suggests that a similar pattern can be observed in the dACC during performance of attention tasks similar to the flanker task (Blasi et al., 2005). One limitation here is the lack of a full replication of the behavioral pattern in the fMRI cohort alone (see Table A1). It should be noted, though that the sample size of the fMRI experiment was smaller than that of the behavioral experiment and therefore possibly underpowered for detection of genotype-related differences in behavior. Brain activity phenotypes have been suggested to be more directly related to the molecular and cellular effects of genetic variations and might thus be more readily detectable in smaller samples (Mier et al., 2010). Therefore, we tentatively suggest that the activation pattern in the dACC might to some extent mirror the behavioral pattern, although caution is warranted. This does, on the other hand, not explain why there was no clear genotype-related ACC activation difference in the PUN condition. One explanation for this observation could be that aversive reinforcement might be more likely to engage other neuromodulatory systems, like the serotonergic system (Daw et al., 2002) in addition to the dopaminergic system, which might reduce the overall influence of genetically mediated differences in dopaminergic neurotransmission during PUN trials.

In addition to the interaction effect in the dACC, genotype-related differences in neural activity patterns included increased activation of the anterior insula in A1 carriers, and *post-hoc* analyses employing confidence interval estimation and masking further suggested that this genotype-related activation difference was largely attributable to the motivated trials. The insula has been commonly found to co-activate with the striatum during reward prediction errors and reward anticipation (for a review, see Diekhof et al., 2012), although some studies argue that insula-dependent processing of cues and prediction errors is particularly critical for the prediction of losses (Palminteri et al., 2012; Metereau and Dreher, 2013) and negative choices (Knutson et al., 2007). Previous studies have demonstrated extensive dopaminergic innervation of the insula (Seamans and Yang, 2004), and the insula also shows substantial structural and functional connectivity with the striatum (de Wit et al., 2012; Palminteri et al., 2012; Ye et al., 2011). Expression of D2 receptors, though, is sparse in the insula where the D1 receptor is the predominant dopamine receptor subtype (Hurd et al., 2001). Considering the high levels of D2 receptor expression in the striatum relative to cortical structures, including the insula, it seems somewhat counterintuitive why a genotype-dependent modulation of motivational processing was observed in the insula rather than the striatum where a more complex interaction of task, genotype, and motivation was observed instead. One possible explanation would be that insula activity during motivational processing might be affected by reduced presynaptic D2 autoreceptor density in A1 carriers. In line with this notion, Laakso et al. (2005) observed higher striatal dopamine synthesis capacity in A1 carriers, which they attributed to reduced D2-mediated autoinhibition of dopaminergic terminals in the striatum. Moreover, pharmacological stimulation of D2-type receptors by pramipexole during reward anticipation has been shown to elicit increased activation of the ventral striatum during reward anticipation, which is accompanied by increased functional connectivity between the striatum and the insula (Ye et al., 2011). We tentatively suggest that the parallel reduction of postsynaptic D2 receptors and increase release of dopamine from presynaptic sites in A1 carriers might result in increased dopaminergic action outside the striatum, as also proposed by Stelzel et al. (2010), who suggested that adaptively increased dopamine signaling in A1 carriers might evoke a more pronounced gating signal that facilitates PFC-dependent updating processes during task switching.

It must be seen as a limitation of our study that our results do not allow to make a direct connection between the increased motivation-related insula activity in A1 carriers, which could be observed across motivated conditions, including COM trials, and the complex behavioral pattern in which the different motivation conditions showed non-linear genotype-related differences. Constituting a key structure of the human salience network (Cauda et al., 2011), the anterior insula has been implicated in focal attentional processes as well as in goal-directed behavior (Dosenbach et al., 2007; Nelson et al., 2010), we therefore tentatively suggest that the increased anterior insula activation in the A1 carriers might reflect an increased recruitment of stimulus-responsive attentional resources in the motivated trials, although the relationship between the increased insula activation and the observed behavioral pattern remains, as of now, subject to speculation and needs to be addressed by future studies.

### Potential molecular mechnanisms underlying the effects of DRD2/ANKK1 TaqIa genotype

Although the TaqIA polymorphism was initially identified during the localization of the DRD2 gene to human chromosome 11q22-23 (Grandy et al., 1989), it has subsequently been pointed out that the SNP is in fact located 10 kb downstream of the DRD2 termination codon on 11q23.1, within coding region of the adjacent ankyrin repeat and kinase domain containing 1 (ANKK1) gene (Dubertret et al., 2004; Neville et al., 2004). Subsequent genetic association studies have since suggested that other genetic variations of ANKK1 might also be associated with addiction disorders (for a review see Ponce et al., 2009). As the DRD2 and ANKK1 gene are closely linked (Neville et al., 2004; Ponce et al., 2009), it has been suggested that genetic variations in linkage disequilibrium (LD) with TaqIA might explain the observed relationship between the SNP and alterations of human dopaminergic neurotransmission. Indeed the DRD2/ANKK1-TaqIA polymorphism is in LD with several polymorphisms on the DRD2 gene (Duan et al., 2003; Ritchie and Noble, 2003; Fossella et al., 2006). Particularly the C957T polymorphism (rs6277) has received considerable attention as it is in LD with TaqIA and affects stability of the DRD2 mRNA (Duan et al., 2003). However, evidence from *in vivo* D2 receptor binding studies is not conclusive and also in apparent conflict with the *in vitro* data (Hirvonen et al., 2004, see also erratum by Hirvonen et al., 2004, 2009a,b). On the other hand, the TaqIA polymorphism, despite being located on the ANKK1 gene, has been repeatedly associated with reduced striatal D2 receptor density in A1 carriers as evident from three *post mortem* studies (Noble et al., 1991; Thompson et al., 1997; Ritchie and Noble, 2003) and two out of three conducted *in vivo* binding studies (Pohjalainen et al., 1998; Laruelle et al., 1998; Jonsson et al., 1999). Moreover, the A1 allele has been associated with increased striatal dopamine synthesis, presumably due to reduced expression of presynaptic autoinhibitory D2 receptors, whereas no association was found between C957T and dopamine synthesis capacity (Laakso et al., 2005). In line with these findings, Stelzel et al. (2010) reported a generally increased striatal BOLD signal in A1 carriers. As striatal BOLD signal has been shown to correlate with dopamine release (Schott et al., 2008), this increased striatal activation might be related to higher presynaptic dopaminergic activity in A1 carriers.

In light of the converging evidence that TaqIA seems to be most reliably associated with lower D2 receptor density further investigations directed at the interaction of DRD2 and ANKK1 is warranted. The predicted ANKK1 protein is an unselective serine/threonine and tyrosine kinase with 11 ankyrin repeats located at the C-terminal end. TaqIA is located in exon 9 of the ANKK1 gene and leads to a glutamate to lysine substitution in the 11th ankyrin repeat. While a direct interaction of DRD2 and ANKK1 has not yet been confirmed, the ontogenetic pattern of ANKK1 expression strongly resembles that of DRD2 and shows upregulation after D2 receptor stimulation by apomorphine (Hoenicka et al., 2010). Strikingly, a genetic variation in close LD with TaqIA, the ANKK1 Ala239Thr polymorphism differentially modulates constitutive and apomorphine-induced ANKK1 expression *in vitro* (Garrido et al., 2011). While D2 receptor-dependent regulation of ANKK1 expression is therefore likely, future research is required to establish whether ANKK1 in turn can also regulate DRD2 expression.

### Limitations and directions for future research

A key limitation of our study is the relatively small sample size, particularly with respect to the behavioral results that reached significance in the behavioral study alone and in the combined cohort, but not in the fMRI experiment alone. Therefore, relating the behavioral and fMRI data to each directly remains to some extent speculative. Another limitation is that, while our results are generally in line with previous studies that have demonstrated effects of DRD2 TaqIA genotype on motivational processes and EFs, one must consider that genetic variations within the dopaminergic system do not exert their effects in isolation. Regarding the flanker task, a human electrophysiological study could demonstrate relatively general effects of a DRD4 genetic variation on error processing, with a further modulation by COMT genotype specifically during stop-signal errors. While, in the present study, we could replicate previous observations of (inefficient) increased prefrontal activation in Val homozygotes (Meyer-Lindenberg et al., 2006), the sample size did not allow us to systematically investigate the combined effects of COMT and DRD2 genetic variations. Future studies should thus further consider the possibility of both additive (Bertolino et al., 2006) and non-linear (Yacubian et al., 2007) gene-gene interactions within the dopaminergic system on human cognitive and motivational processing.

## Conclusions

Taken together our results provide further evidence for a modulation of PFC-dependent EFs by motivational salience. Behaviorally, motivation was associated with overall RT reduction across flanker conditions. At a neural level, we observed a motivation-related reduction of DLPFC activation specifically during the incongruent vs. congruent flanker trials, suggesting that motivational salience might result in higher processing efficiency. A genetic variation that has previously been linked to striatal dopamine D2 receptor availability did not affect overall performance as indexed by RTs, but instead, showed a complex interaction with motivation on interference effects. A1 carriers with presumably lower D2 expression showed (at least nominally) improved interference processing during rewarded trials, while A2 homozygotes primarily benefitted from the combination of appetitive and aversive reinforcement. At a neural level, a compatible pattern was observed in a complex genotype by task interaction in the dACC. Additionally, A1 carriers showed an increased neural response of the anterior insula, an effect mostly driven by motivationally salient stimuli. These findings are in line with previous research linking prefrontal dopamine to performance of EFs, possibly following an inverse U-shaped function.

### Conflict of interest statement

The authors declare that the research was conducted in the absence of any commercial or financial relationships that could be construed as a potential conflict of interest.

## Appendix

**Table A1:** **Behavioral data (*t*-statistics), separated by experiment**.

	**A1+**		**A1−**	
**Behavioral experiment**	***t*_(23)_**	***p***	***t*_(21)_**	***p***
REW vs. NEU	−1.89	**0.036^*^**	0.45	0.331
PUN vs. NEU	−1.45	0.080	1.51	0.073
COM vs. NEU	−1.12	0.137	−1.01	0.163
REW vs. PUN	−0.51	0.614	−1.27	0.220
COM vs. REW	1.08	0.147	−1.88	**0.037^*^**
COM vs. PUN	0.48	0.319	−3.74	**<0.001^*^**
**fMRI experiment**	***t*_(14)_**	***p***	***t*_(16)_**	***p***
REW vs. NEU	0.41	0.345	0.11	0.457
PUN vs. NEU	−0.16	0.439	0.07	0.471
COM vs. NEU	0.61	0.277	−0.77	0.226
REW vs. PUN	0.60	0.559	0.04	0.971
COM vs. REW	0.20	0.423	−0.82	0.213
COM vs. PUN	0.81	0.215	−0.97	0.173

Results of post hoc paired T-tests testing for effects of the motivation conditions on the congruency effect of reaction times, separated by genotype group and experiment. All p-values are one-tailed, except for the REW vs. PUN contrast for which we had no directed hypothesis. NEU, neutral condition; REW, reward condition; PUN, punishment condition; COM, combined reward and punishment condition. Note: As there was no genotype by motivation interaction in the fMRI experiment alone, all t- and p-values are displayed for illustrative purpose only and printed in grey.

* p < 0.05.
